# hPSC-Derived Astrocytes at the Forefront of Translational Applications in Neurological Disorders

**DOI:** 10.3390/cells13110903

**Published:** 2024-05-24

**Authors:** Vukasin M. Jovanovic, Kendall T. Mesch, Carlos A. Tristan

**Affiliations:** Stem Cell Translation Laboratory (SCTL), Division of Preclinical Innovation (DPI), National Center for Advancing Translational Sciences (NCATS), NIH, Rockville, MD 20850, USAcarlos.tristan@nih.gov (C.A.T.)

**Keywords:** hPSC, astrocyte, disease modeling, drug discovery

## Abstract

Astrocytes, the most abundant glial cell type in the brain, play crucial roles in maintaining homeostasis within the central nervous system (CNS). Impairment or abnormalities of typical astrocyte functions in the CNS serve as a causative or contributing factor in numerous neurodevelopmental, neurodegenerative, and neuropsychiatric disorders. Currently, disease-modeling and drug-screening approaches, primarily focused on human astrocytes, rely on human pluripotent stem cell (hPSC)-derived astrocytes. However, it is important to acknowledge that these hPSC-derived astrocytes exhibit notable differences across studies and when compared to their in vivo counterparts. These differences may potentially compromise translational outcomes if not carefully accounted for. This review aims to explore state-of-the-art in vitro models of human astrocyte development, focusing on the developmental processes, functional maturity, and technical aspects of various hPSC-derived astrocyte differentiation protocols. Additionally, it summarizes their successful application in modeling neurological disorders. The discussion extends to recent advancements in the large-scale production of human astrocytes and their application in developing high-throughput assays conducive to therapeutic drug discovery.

## 1. Introduction

Astrocytes were first described in the 19th century as a homogenous cell population and believed to have a passive and supportive role for neuronal function [1]. Golgi and Cajal performed a more extensive morphological characterization of astrocytes and discovered the heterogeneity of these cells [2]. Among mammals, human astrocytes exhibit distinct features, including notably larger volumes, increased synaptic interactions, enhanced calcium propagation, and double the number of morphological subtypes compared to astrocytes found in rodents [3,4,5,6]. Astrocytes regulate CNS homeostasis by maintaining the BBB, setting an ionic tone, recycling neurotransmitters, and functioning as a source of neuronal energy and metabolism regulation. They are also crucial in the development and formation of the BBB, dendritic spine growth, synapse formation and pruning, neuronal migration, and axon guidance [7,8,9]. Most astrocytes contact the vasculature and may readily respond to changes in peripheral blood circulation [5,10]. During neuroinflammation, astrocytes respond to microglia-secreted inflammatory cytokines and become reactive [11]. These reactive astrocytes have been further subcategorized as neurotoxic, responsive to activated microglia [12], and neuroprotective [13]. The neurotoxic effect of reactive astrocytes is mediated via the secretion of toxic long-chain saturated lipids [14], while the neuroprotective astrocytic function can be mediated with an increase in perinuclear cAMP and soluble adenylyl cyclase [13]. However, the field has transitioned away from the binary classification of reactive astrocytes in disease states [15]. It has become increasingly evident that reactive astrocytes exhibit a far greater diversity with distinct molecular states observed in different disease models [16,17,18]. Due to their numerous physiological functions, astrocytes exhibit a multifaceted role in neurological disorders. As a causative or contributing factor, astrocytes have been associated with neurodevelopmental disorders such as ASD [19], a rare neurogenetic leukodystrophy known as Alexander disease (AxD) [20], and more prevalent neurodegenerative disorders such as AD [21,22,23], PD [24], ALS [25], MS [26,27], and others [28,29]. Due to being a long-neglected component of the CNS, the full functionality of human astrocytes is still enigmatic; however, their role in normal physiology and pathophysiology is slowly being uncovered.

The use of murine astrocytes for modeling mammal astrocytic properties has successfully uncovered the trophic, phagocytotic, and immuno-reactive functional characteristics of astrocytes that have since been replicated in human astrocytes. However, the differences in transcriptomic profiles, subtypes, and overall complexities between primary human and murine astrocytes highlight a significant challenge in relying on other mammals for the modeling of human disease [5].

Protoplasmic, the most numerous structural subtype, and fibrous astrocytes are common between humans and rodents. However, in humans, there are additional subtypes of astrocytes that are not present in the brains of rodents or even non-human primates [5]. Human astrocytes are notably larger and more structurally complex compared to those in rodents. Human protoplasmic astrocytes also cover larger areas, facilitating extensive synaptic interactions—up to approximately 2 million synapses per astrocyte. Additionally, adult human astrocytes respond nearly five times faster to ATP and glutamate signals [5]. Adult human astrocytes, unlike adult mouse astrocytes, robustly respond to varying concentrations of glutamate [30]. The transcriptomic profiles of mouse astrocytes exhibit a mere 30% overlap with human astrocyte enriched genes. Moreover, there are many human astrocyte genes and long non-coding RNAs that lack orthologs in the mouse brain, underscoring considerable differences between the species [30]. While the additional human astrocytes subtypes may contribute to this lack of genetic overlap, further investigation is needed.

This review will focus on analyzing the existing literature pertaining to in vivo astrocyte development, their cellular and functional heterogeneity, and how these characteristics are reflected across hPSC-derived in vitro human astrocyte models. To advance the utility of hPSC models in preclinical and clinical applications, increased standardization across differentiation methods must be established. This will promote a deeper understanding of the developmental stages of the derived cells, their functional characteristics, and assess whether a specific differentiation method has the scalability required for translational applications.

## 2. In Vivo Development and Heterogeneity of Astrocytes

Most studies delving into astrocyte development or astrogenesis have been conducted in animal models [31,32]. More recently, with the advent of single-cell sequencing technologies, cell populations have been profiled across human fetal brain development [33,34]. However, most studies have focused extensively on mapping the early neuronal specification and developmental trajectories of neurogenesis, while gliogenesis still remains understudied.

**Figure 1:**
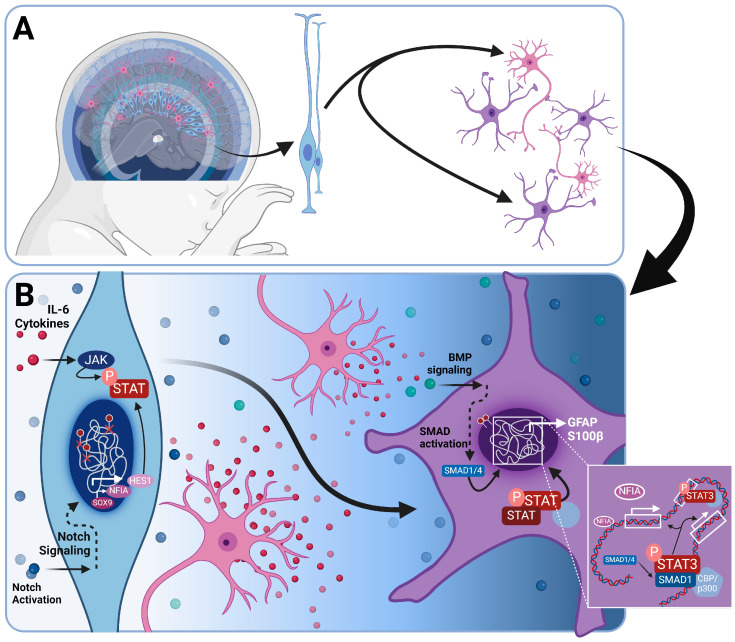
Gliogenic switch during brain development. (**A**) During early brain development, RGCs, depicted in blue, emerge as the predominant cell type. They possess the ability to generate neurons (depicted in pink) and astrocytes (depicted in purple) in a temporally segregated manner. Moreover, aside from astrocytes and neurons, RGCs also generate oligodendrocytes and NG-2 glia, which are not illustrated in this image. (**B**) Intrinsic cell programs during the initiation of the gliogenic switch in RGCs are in part controlled by the activation of JAK/STAT by IL-6 family cytokines (depicted in red dots) secreted by nascent neurons (depicted in pink) and NOTCH ligands (blue dots) activating the NOTCH signaling pathway in RGCs. Additional factors implicated in astrogenesis include SOX9, which is closely associated with NFIA activity. Abbreviations used in this figure are defined in the list of abbreviations.

**Figure 2:**
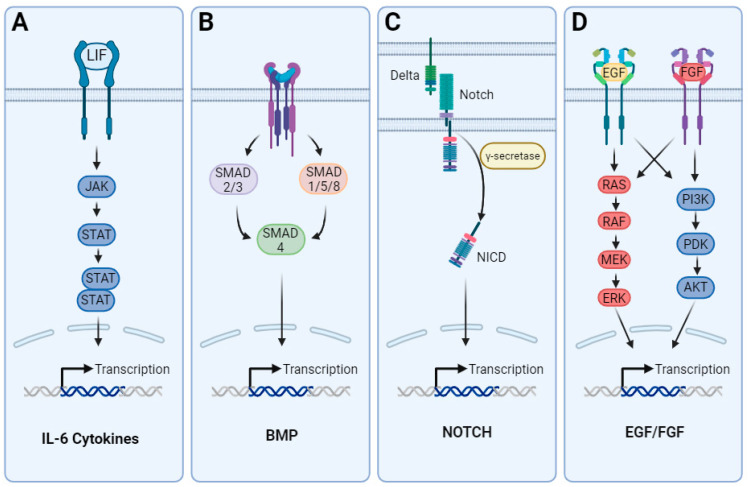
Transcription regulators of key genes in astrogenesis. (**A**) Cytokine receptor and its effectors. Binding of ligands from the IL-6 family of cytokines (e.g., LIF, ONC-M, CT-1) brings the receptor associated janus kinases into contact, leading to their phosphorylation and binding to STATs. Activated STATs translocate to the cell nucleus as homodimers or heterodimers and regulate the transcription of target genes. (**B**) Schematic example of BMP receptor and its effectors in the canonical BMP signaling pathway. This signal transduction cascade is initiated by binding to cell surface receptors and forming a heterotetrameric complex comprised of two dimers of type I and type II serine/threonine kinase receptors. The constitutively active type II receptor transphosphorylates the type I receptor when a heterotetrameric complex is formed. By doing so, the type I receptor is activated, which allows receptor-regulated Smads, or R-Smads, to be phosphorylated. Smad1, Smad5, and Smad8 are the R-Smads that participate in BMP signaling (Smad1/5/8). Following their association, R-Smads bind to co-mediator Smad (co-Smad) Smad4, and this complex moves to the nucleus where it acts as a transcription factor to control the expression of genes in conjunction with coactivators and corepressors. Smad6 and Smad7 (Smad6/7), also known as inhibitory Smads (I-Smads), are involved in the signaling pathway feedback inhibition. (**C**) The transmembrane proteins Delta and Serrate (called Jagged in mammals) are NOTCH receptor ligands. Two proteolytic cleavage events in the NOTCH signaling cascade are facilitated by ligand–receptor interaction. ADAM-family metalloproteases catalyze the first cleavage, while γ-secretase, an enzyme complex, mediates the second. The NOTCH intracellular domain (Nicd), which is released by the second cleavage, moves into the nucleus and works to induce transcription with the DNA-binding protein CSL and its co-activator Mastermind. (**D**) EGF receptor traditional function involves activating several downstream signaling cascades to convey extracellular mitogenic signals, such as transforming growth factor-α (TGF-α) and EGF. Among them are signaling modules involving phosphatidylinositol-3 kinase, Ras, and phospholipase C-γ. Pathways primarily involved in FGF signal transduction control various cell processes. Cell proliferation and differentiation are regulated by the RAS/MAP kinase pathway, which is triggered by the creation of an FRS2 complex. The creation of an FRS2 complex also initiates the PI3/AKT pathway, which controls cell survival and destiny determination. Ultimately, DAG and IP3 are created upon PLCγ binding to the active FGFR, activating PKC. The PLCγ pathway affects the adhesion, migration, and shape of cells. Due to overlapping signaling effectors, these two pathways exhibit significant crosstalk. While factors driving astrogenesis significantly differ from those involved in neurogenesis, the developmental heterogeneity of astrocytes is largely dependent on the same patterning mechanisms that drive the spatial distribution and identity of neurons across the CNS [64]. Recently, single-cell spatial transcriptomics analyses of rodent cortex suggested an intricate layering of astrocytes instructed by cortical neuronal layering during early postnatal development [65]. Therefore, the demonstration of developmental heterogeneity among astrocytes prompts inquiries into their functional diversity that may depend on the specific brain region or the neural circuitry to which they belong [66]. Acquiring a deeper understanding of the specialized astrocyte populations could lead to new therapeutic modalities for the treatment of neurological disorders. Abbreviations used in this figure are defined in the list of abbreviations.

In the early stages of brain development, the neuroepithelial progenitor pool expands and starts forming RGCs, a type of elongated bipolar cell, from which the majority of CNS cellular subtypes emerge. RGCs also provide a scaffold for migrating newly born neurons (Figure 1A) [35,36]. Neurogenic precursors primarily originate as RGC progeny during asymmetric division within the ventricular zone of the developing neural tube [37,38]. As the wall of the neural tube expands, the subventricular zone emerges with neurogenic basal progenitors that divide symmetrically and propagate neuronal production [39,40]. This neurodevelopmental phase is known as neurogenesis. Human astrogenesis occurs at late developmental stages when the remaining RGCs undergo a switch from largely producing neuroblasts to largely producing glial precursors [41]. In the mouse cortex, glioblasts resulting from the asymmetric division of radial glia are observed between embryonic days 16 and 18 (E16–E18) [42]. These proliferative glial progenitors undergo radial migration and subsequently give rise to multiple clusters of astrocytes within the same cortical column. In non-human primates, such as rhesus monkeys, gliogenesis occurs mid-gestation between embryonic days 65 and 90 [43]. In humans, the exact timing of the gliogenesis peak varies depending on the brain region. Most studies report an acceleration in gliogenesis between gestational weeks 20 through 40 [44,45,46]. Nascent neurons release signaling factors, including the IL-6 cytokine family members like CT-1, that bind to signaling co-receptors LIFR-beta and gp130, initiating the JAK/STAT signaling cascade (Figure 1B) [47,48]. NPCs in mice lacking LIFR or gp130 exhibit significant deficiencies in initiating astrogenesis, leading to a dramatic reduction in the population of astrocytes [49]. Other activators for LIFR-beta and gp130 include CNTF and LIF, which are additional members of the IL-6 cytokine family. CNTF is found to strongly induce the phosphorylation of JAK1, STAT1, and STAT3 in NPCs, leading to an increase in GFAP positive cells [50] (Figure 1B). However, neural progenitors isolated from rodent embryonic neural tubes at the peak of neurogenesis do not undergo astrogenesis in response to LIFR activation, suggesting that additional intrinsic cues are required for the initiation of this switch [51]. During neurogenesis, gliogenesis is actively inhibited by epigenetic mechanisms and the inhibition of activating transcription factors. The effective inhibition of STAT activation by Neurogenin-1 during active neurogenesis prevents the onset of gliogenesis in NPCs [52]. This inhibition is reinforced by the segregation of activator complexes, such as Smad1/CBP/p300 away from the regulatory regions of glial-specific genes [53]. BMPs have diverse effects on neural stem cell fate choice [54] and have been extensively associated to the initiation of astrogenesis in NPCs [55]. In addition, the overexpression of BMP4 at late timepoints of cortical development leads to an increase in the number of astrocytes alongside a decrease in the number of oligodendrocytes. This observation suggests that BMP signaling plays an important role in the advanced stages of astrocyte specification [56]. The loss of neurogenic capacity of RGCs has also been attributed to exposure to Notch ligands, which are expressed on newly born neuroblasts [57] (Figure 1B). An increase in Notch signaling results in an inhibition of neurogenesis [58] and upregulation of NFIA [59], which in contrast allows the propagation of gliogenesis and maintenance of the RGC progenitor pool for the production of glia. EGF and FGF2 (bFGF), known mitogens, have also been implicated in the onset and propagation of gliogenesis. Abnormal astrocyte development has been observed in mice deficient in EGFR [60], while the exogenous administration of EGF induces gliogenesis in embryonic neural stem cells [61]. FGF-2 signaling during the gliogenic switch activates the MAPK pathway and inhibits neurogenesis [62]. The activation of FGF signaling during astrogenesis dramatically increases the number of astrocytes in the cerebral cortex [63]. Signaling cascades for each of the aforementioned pathways are depicted in Figure 2.

## 3. Differentiation of Astrocytes from hPSCs

Decades of research on brain development in animal models have paved the way and provided the basis for the establishment of neural differentiation protocols from hPSCs. The differentiation of astrocytes from hPSCs has proven to be particularly challenging with varying efficiencies across different protocols. Predominantly, these protocols can be subcategorized into (i) chemically undefined [67,68], (ii) chemically defined [69,70] and (iii) doxycycline-inducible overexpression systems [71,72,73,74]. The incorporation of xenogeneic components, like FBS and serum-based commercially available media, has been a relatively simple way to enhance the cell culture environment, prevent neurogenesis, and promote gliogenesis in hPSC-derived NPCs [67,68]. The use of serum comes with multiple caveats, as exposure to serum can induce divergent and irreversible genetic and morphological differences as compared to healthy in vivo astrocytes [30]. In addition, astrocyte differentiation media and serum replacement systems available commercially, like knockout-serum replacement, often contain proprietary ingredients. These ingredients can affect the consistency and performance of derived astrocytes across different batches. Despite this knowledge, multiple hPSC-differentiation protocols for astrocytes within the past decade have taken advantage of FBS or proprietary media formulations which may have consequences on the translational value of these cells (Table 1). Due to the protracted developmental trajectory in humans when compared to other mammals, a benchmark astrocyte differentiation protocol was developed to model the entire gliogenic switch. This protocol utilized mitogen-based growth factors, EGF and FGF2, to drive expansion and generate immature astrocytes in a period of 6 months [69,75]. The derived cells possessed molecular and functional characteristics similar to human fetal astrocytes and maintained an astroglial identity in vivo when transplanted into the brains of mice. Subsequent protocols have been developed with reduced differentiation timeframes that generate functional fetal-like astrocytes in 30 to 50 days [67,70]. Recent studies have suggested that the expression of NFIA in neural stem cells may function as the main driver of the gliogenic switch, leading to the acquisition of glial competency in these cells. Moreover, NFIA overexpression induces the gliogenic switch within 5–6 days; however, protracted culture and the administration of exogenous factors were required to induce GFAP expression [71]. Our team developed a scalable, stepwise, and controlled approach to induce NFIA with the use of exogenous factors within 21 days of differentiation from the hPSC stage [70]. By using recombinant proteins to target the JAK/STAT and NOTCH signaling pathways, this protocol achieves the stepwise generation of neural rosette-forming FABP7^+^ RGCs within 7 days, NFIA^+^ astroglia progenitors by day 21, CD44^+^NFIA^+^ immature astrocytes at day 30 and GFAP^+^ astrocytes by day 50. Transcriptomic profiling and chromatin landscape analyses as well as direct comparisons to human fetal astrocytes, indicate that these hPSC-derived astrocytes are at a fetal-like developmental stage. However, the accessibility of the GFAP promoter at later stages suggests the attainment of a more mature identity. Importantly, this work also suggested the translational utility of fetal-like astrocytes, as these cells were used for AxD modeling and mouse brain grafting experiments [70].

Complex culture methods, such as long-term 3D cultures of cerebral cortical spheroids, coupled with immunopanning to isolate astrocytes, have demonstrated increased expression levels of mature astrocyte markers, such as AQP-4 and ALDH1L1, alongside the downregulation of markers associated with cell cycle progression and proliferation [76]. In addition, these astrocytes exhibited functional maturation as the phagocytic activity, a fetal-like astrocyte characteristic, of these cells declined with increased time in 3D culture [76]. However, the scalability of 3D approaches remains challenging, and reliable markers for the immunopanning and isolation of astrocytes upon the dissociation of these spheroids is required for translational applications [77]. Recently, the prolonged culture of 3D spheres in spinner flasks was suggested as an approach to generate a large number of astrocytes upon dissociation [78].

NFIA, NFIB, and SOX9 transcription factor overexpression using CRISPR/Cas9 edited DOX-inducible cell lines has been proposed for the rapid induction of astrocyte identity in hPSCs [71,72,73]. The co-expression of NFIB and SOX9 facilitates the induction of astrocyte specification within 21 days. These cells expressed canonical astrocyte markers S100B, GFAP, and VIM in high percentages (~80–96%) and were used for mouse brain grafting and AxD modeling [72]. A comprehensive summary of differentiation time, culture methods, compounds used, as well as the scalability of the mentioned approaches is listed in Table 1. Functional characteristics, including the maturation stage of the astrocytes derived from different protocols, multi-omics characterization datasets and cell-based assays used for functional astrocyte characterization are listed in Table 2.

In addition to these approaches, transdifferentiation or the direct conversion methods of somatic cell types into other cell types have been established. These methods employ the transduction of a single or a set of transcription factors to induce the conversion of terminally differentiated cells from one cell type to another. Various studies have reported the transdifferentiation of cells to generate neurons [79], cardiomyocytes [80], hepatocytes [81], macrophages [82], and astrocytes [83], among others. In contrast to the generation of somatic cell types from hPSCs, transdifferentiation preserves the epigenetic marks and age of the source cell, resulting in the retention of age-related characteristics and maturity that may facilitate the establishment of models for diseases associated with aging [84]. Although transdifferentiation methods are becoming increasingly effective, they have two major shortcomings compared to the derivation of cells using hPSCs in terms of translational applications. While hPSCs offer a renewable source of patient-derived cells, the scalability of transdifferentiation approaches is limited by the availability of the primary cell source. Another significant concern is the genomic integration of transgenes, which can lead to genetic abnormalities that drive transformation, induce proliferation, and affect functional gene expression. Therefore, alternative integration-free approaches are continuously being developed.

## 4. hPSC-Derived Astrocytes for Modeling Rare and Common Neurological Disorders

Despite the challenges in hPSC differentiation to astrocytes, and the fetal-like developmental stage of the derived astrocytes, hPSC-derived astrocytes are in high demand for the modeling of neurological disorders. AxD is a rare neurogenetic disorder, belonging to the family of diseases termed leukodystrophies. AxD is caused by a mutation in the GFAP gene and predominantly affects astrocytes in the CNS, leading to misfolded protein accumulations known as Rosenthal fibers [85]. Modeling AxD using patient iPSC-derived astrocytes have led to novel findings about the pathomechanisms of this disease. AxD astrocytes were found to inhibit OPC differentiation into oligodendrocytes due to the decreased proliferation of OPCs [86]. Furthermore, other studies found that astrocytes affected by AxD exhibit TDP-43 aggregation [70] and an increased secretion of inflammatory cytokines [87]. These findings highlight the complex pathophysiology of AxD and underscore the translational value of such hPSC-derived models to elucidate disease mechanisms and develop novel treatments.

Exaggerated calcium (Ca^2+^) signaling fluctuations have recently been demonstrated in hPSC-derived astrocytes from ASD patients [88]. When transplanted into mouse brains, these astrocytes have caused reduced neuronal spine density and even induced repetitive behaviors in chimeric animals, which is a hallmark symptom of ASD [88]. In a set of separate experiments, hPSC-derived astrocytes from ASD patients caused neuronal defects when co-cultured with healthy hPSC-derived neurons [89]. These defects included delayed synaptogenesis and a reduced spontaneous firing rate, which were attributed to the increased secretion of inflammatory IL-6 by ASD astrocytes [89].

Glial cell types have also been heavily implicated in the onset and propagation of complex pathophysiology in neurodegenerative diseases. AD patient-derived astrocytes exhibit reduced morphological heterogeneity [90]. The APOE4 allele is the single most significantly associated genetic risk variant for sporadic cases of AD. Isogenic cell lines are a powerful tool to gain insight into the biological processes associated with specific genetic variations between paired cell lines. The generation of these isogenic pairs is predominantly accomplished through targeted gene-editing techniques including CRISPR/Cas9, transcription activator-like effector nucleases (TALEN) and zinc finger nucleases [91,92,93]. There are mainly two types of isogenic cell lines: one is established utilizing a normal or healthy control cell line in which a specific genetic modification is made to generate an abnormal or mutant cell line, and the other is established from a patient-derived cell line in which a specific genetic mutation is corrected. Isogenic hPSC lines establish an avenue for the direct association of gene function to cellular processes and disease susceptibility in a fixed genetic background. Since most APOE is produced by astrocytes in the CNS, a recent study used the CRISPR/Cas9 system to generate iPSC lines carrying homozygous APOE4 alleles and discovered that astrocytes differentiated from such iPSC lines exhibit reduced APOE expression, altered cholesterol metabolism, and the impaired uptake of amyloid beta [94]. In contrast, rare early onset familial AD cases are associated with mutations in PSEN1/2. Another recent study used hPSC lines derived from patients with rare early onset familial AD carrying a PSEN1 mutation to generate astrocytes. These astrocytes also displayed profound metabolism impairment leading to oxidative stress, reduced lactate production, and impaired metabolic support to neurons [95].

hPSC-derived astrocyte models have also been leveraged for PD modeling. In one such study, hPSC lines derived from patients carrying familial *LRRK2* mutations were differentiated into S100B, GFAP, and GLT-1 expressing astrocytes and co-cultured with ventral midbrain-like hPSC-derived dopaminergic neurons. Patient-derived astrocytes had accumulated alpha synuclein and induced the neurodegeneration of healthy dopamine neurons via alpha synuclein accumulation when co-cultured [95]. In an independent study modeling BBB formation on a 3D chip, *LRRK2* patient-derived astrocytes were found to produce increased amounts of inflammatory cytokines and displayed altered angiogenic stimulus secretion resulting in failure to support BBB formation [96].

Extensive studies have been conducted using hPSC-derived astrocytes from patients with ALS and ALS overlapping with frontotemporal dementia (FTD) [97,98,99,100]. There are ongoing clinical cell therapy studies in ALS patients using hPSC-derived astrocytes and GDNF-secreting glial precursors [101].

hPSC-derived astrocytes from MS patients have shown an altered response to inflammatory cytokines and were associated with reactive astrogliosis and increased CNS access of peripheral immune cells into MS lesions [102,103].

Furthermore, developmental perturbations in schizophrenia were modeled using hPSC-derived astrocyte models [104]. hPSC-derived astrocytes from schizophrenic patients demonstrated altered calcium signaling and reduced glutamate uptake capacity [104]. hPSC-derived glia progenitors from patients with juvenile onset schizophrenia demonstrated delayed astrocyte maturation when transplanted into mouse brains [105]. These findings collectively indicate that astrocytes play a significant role across a spectrum of both common and rare neurogenetic, neurodevelopmental, neurodegenerative, and neuropsychiatric disorders.

## 5. Advancements in Biomanufacturing of Human Astrocytes for Disease Modeling and Drug Screening

The advent of iPSC technology has led to the development of numerous differentiation protocols to generate a renewable source of human somatic cell types including those that were previously difficult or impossible to obtain. These innovations have revolutionized the fields of translational research and developmental biology by providing unprecedented opportunities to recapitulate human disease and embryonic development ‘in a dish.’ Despite these advances, many reported protocols rely on laborious and time-consuming manual processes that inherently limit their scalability and reproducibility. The integration of recent advances in iPSC and automation technologies has created a pivotal point in translational research. This convergence offers remarkable potential to address previous biomanufacturing bottlenecks for disease modeling, therapeutic development, and regenerative medicine [106]. The first reported efforts to automate hPSC culture focused primarily on developing strategies to address labor-intensive maintenance and expansion processes that would reduce variability and technician involvement to generate a reliable source of hPSCs [107,108,109]. Expanding on these efforts, scientists at the New York Stem Cell Foundation were among the first groups to pioneer the combination of several modular robotic platforms for high-throughput iPSC reprogramming, expansion, and differentiation [110]. Here, they report the construction of a modular automated hub composed of eight robotic instrument clusters capable of cell expansion, passaging, cryopreservation, thawing, mycoplasma testing, plating, imaging, quality control, magnetic sorting, cherry picking, embryoid body Scorecard analysis, and the generation of cell pellets for RNA/DNA extraction [93]. This hub of automated platforms can generate iPSC lines with reduced variation in differentiation potential when compared to manually derived lines. The automated medium-exchange module of this platform is then utilized to differentiate iPSCs in a 96-well format to generate definitive endoderm within 3 days and midbrain dopaminergic neurons through a 30-day differentiation protocol.

Similarly, other groups have also reported the use of smaller scale and less expensive automated systems for the reprogramming, maintenance, and quality control of hPSCs that can be readily adopted by most labs. The use of scalable and self-contained robotic liquid handlers with or without a manipulator arm have been demonstrated as an economical alternative to large-scale platforms for maintaining and differentiating adherent hPSC cultures in multi-well plate formats [108,111,112]. However, although these platforms were demonstrated to have the capability to differentiate hPSCs into various cell types, the primary focus of these studies was on iPS cell line development and expansion which has facilitated the establishment of large-scale iPSC repositories from diverse patient and healthy control populations.

Various self-contained robotic liquid handlers have been utilized to culture hPSCs in both monolayer and multi-well formats, enabling high-throughput differentiation into diverse somatic cell types such as neurons, cardiomyocytes, and hepatocyte-like cells [110,113,114]. By employing automated liquid handling systems, researchers can now streamline the differentiation process, optimize protocols, and enhance the reliability of results, thus accelerating translational research.

More recently, interest has begun to shift from iPS cell line development toward the industrial scale biomanufacturing of hPSC-derived cell types for translational applications. At NCATS, the incorporation of robotic biomanufacturing with iPSC technologies has allowed us to achieve remarkable advancements in the large-scale production of cortical neurons, cardiomyocytes, hepatocytes, and peripheral sensory neurons for disease modeling and drug screening [115,116]. As mentioned, in 2023, we developed a controlled, efficient, and xeno-free protocol for the rapid generation of hPSC-derived astrocytes [70]. This accelerated and chemically defined differentiation protocol first yields proliferative, multipotent RGCs that are then directly differentiated to GFAP expressing astrocytes, notably bypassing neurogenesis. The automated culture of these proliferative RGCs on the CompacT SelecT robotic cell culture platform streamlined otherwise laborious culturing steps to generate astroglia progenitors and astrocytes. Adherent cells, such as those described above, are grown on planar flasks, and production limits are directly associated to the surface area available for cell growth with yields ranging 52–472 million cell per flask or 10–50 billion per large-scale run. To our knowledge, this work is the only reported study to demonstrate an automated workflow for the differentiation of hPSCs into astrocytes [106,115].

## 6. High-Throughput Cell-Based Functional Astrocyte Assays

Investigators have developed high-throughput assays using astrocytes to gain insight into their neurobiological functions within the CNS and various brain pathologies. These investigations have been directed toward establishing robust assays to explore various facets of astrocytic biology, including AQP-4 clustering and the kinetics of calcium signaling, as well as their roles in synaptic development and the reactive astrogliosis process [117,118,119,120]. Despite the valuable insights provided by these assays into disorders such as brain edema, AD, and PD, their utility is limited by their dependence on primary rodent or human astrocytes. These cell sources may not fully recapitulate human physiology and can be challenging to obtain. To overcome these limitations, investigators are increasingly turning to hPSCs as an unlimited and scalable source of diverse brain cell types.

**Figure 3:**
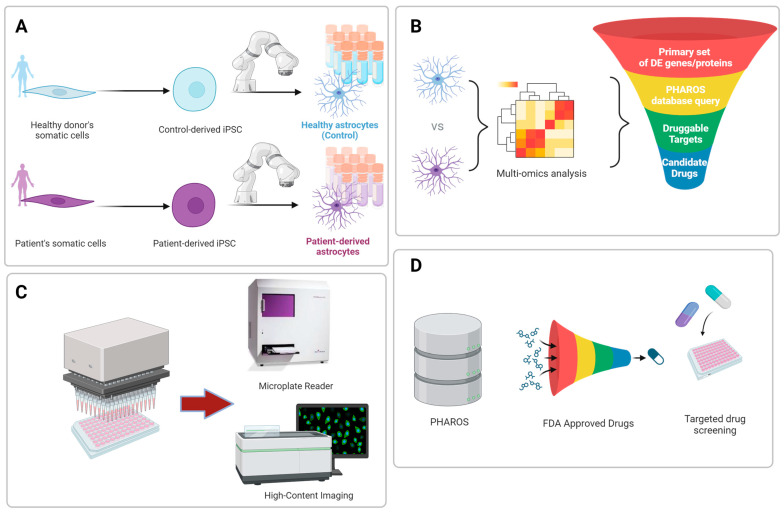
Schematic overview of the translational potential of iPSC-derived astrocytes for modeling neurogenetic disorders and therapeutic drug discovery. (**A**) Generation of patient derived iPSC lines and robotic differentiation into astrocytes. (**B**) Two-step process for druggable target identification involving (i) multi-omics profiling to identify differentially regulated genes/proteins and (ii) druggable genome database query to identify druggable targets. (**C**) Assay development amenable to high-throughput applications. (**D**) Example of drug repurposing. Targeted drug screening using only the set of FDA-approved drugs among the identified drug candidates. Abbreviations used in this figure are defined in the list of abbreviations.

The at-scale production of hPSC-derived cell types, either manually or via automated platforms, has facilitated the development of high-throughput cell-based assays and in vitro models of disease, providing a valuable tool for translational research in drug discovery and therapeutic development. Due to significant biological differences between species, these human cell-based models have the ability to more closely recapitulate in vivo human physiology when compared to existing animal models [121]. Moreover, the use of hPSC-derived cell types, including astrocytes, creates an opportunity to realize precision medicine where patient-derived cells can be utilized to develop personalized treatments specifically tailored to an individual’s genetic background (Figure 3A–D). The field is now beginning to explore such cell-based assays and in vitro models as tools to reduce cost and the use of traditional animal testing, addressing associated ethical concerns. In light of these advances, the FDA Modernization Act 2.0 was signed into law on 29 December 2022 to permit the utilization of alternatives to animal testing, including cell-based assays, such as hPSC derived organoids, organs-on-chips, and advanced artificial intelligence methods [122]. This paradigm shift allows preclinical studies to use these more physiologically robust systems as alternatives to outdated animal testing. When combined with high-throughput screening, these innovative hPSC-derived approaches can reduce cost and accelerate the drug development process.

Drug discovery efforts that target astrocytes have been used to identify compounds that protect them from damaging insults within the CNS. These insults include oxidative stress, which is linked to the development of various neurological diseases. Astrocytes possess multiple antioxidant systems, such as the glutathione system, which have been shown to safeguard neurons against oxidative damage [123,124]. Therefore, the identification of compounds that protect astrocytes from oxidative damage could assist in addressing pathologies of neurological diseases. To this end, a high-throughput and high-content nuclear morphology assay using hPSC-derived astrocytes was developed to identify compounds that protect astrocytes from oxidative damage [125]. Here, a high-throughput screening platform is used to screen about 4100 compounds and approved drugs in a 1536-well format to identify compounds that prevent oxidative stress-induced cell death in hPSC-derived astrocytes. High-content imaging analysis of apoptotic nuclear parameters identified 22 hit compounds with cytoprotective effects in astrocytes. This approach represents another proof-of-concept platform that can be used for neurodegenerative drug discovery.

By coupling the use of newly established hPSC differentiation protocols to generate physiologically relevant human cell types with advances in automation technologies, recent studies have developed improved in vitro models of various human physiological systems that are amenable to high-throughput screening and therapeutic development. For instance, Bassil et al. incorporated the versatility of a liquid handler for use in cell plating, media changes, experimental treatment, and fixation steps with hPSC-derived neurons, astrocytes, and microglia to generate a triculture model of human AD [126]. This triculture model system was demonstrated to develop neuritic plaque formation with surrounding microglia similar to that observed in human AD postmortem brain sections. The integration of this triculture model with the Fluent automation workstation and a high-content imaging system was used to perform proof of concept screening studies that identified several compounds targeting proteins of known mechanisms in AD. This platform was then used to analyze cellular mechanisms of amyloid plaque formation, model AD progression, and evaluate the effects of anti-amyloid beta interventions. Together, this triculture system was capable of recapitulating hallmarks of human AD in a ready-to-screen format.

The development of high-throughput cell-based assays utilizing hPSC-derived astrocytes marks a significant advancement in neurodegenerative disease research and drug discovery. Importantly, in vitro model systems are rapidly evolving and increasing in complexity, aimed at building complete model systems that incorporate multiple components of in vivo biology. This is exemplified by the view of the tetrapartite synapse model that aims to incorporate oligodendrocytes, astrocytes, microglia, and elements of the extracellular membrane to recapitulate the synaptic cleft more closely [127]. However, as in vitro models approach the complexity of in vivo systems, their utility is limited due to cost and throughput constraints. High-throughput screening campaigns using cell-based functional astrocyte assays will provide insights into astrocyte biology, pathophysiology, and their intricate roles in neurological disorders.

## 7. Conclusions

Cells of the central and peripheral nervous system have historically been difficult to obtain due to their intricate and delicate nature as well as the complexity of the environment in which they reside. Challenges in acquiring neural cell types are associated with several factors that include inaccessibility within the body, vulnerability to damage, and their limited regenerative and/or proliferative capacity.

Human hPSC-derived astrocytes are distinct from historically popular animal models, such as murine models used for studying neural diseases and disorders. Additionally, the use of human neural tissue from postmortem human brains for studying human astrocytes involves obtaining highly limited and expensive tissues that can lack the full complexity of astrocytes in vivo.

The transition from reliance on primary cells to utilizing hPSCs, an inexhaustible reservoir of diverse cell types, represents a significant advancement in the evolution of high-throughput cell-based functional assays. Similarly, the incorporation of hPSC-derived brain cell types including astrocytes into increasingly complex 2D and 3D models that more faithfully model human physiology signifies a pivotal point in neurodegenerative disease research and drug discovery. When integrated with patient-derived cells, cell-based functional assays, and high-throughput screening campaigns, these hPSC-derived astrocyte models hold great promise in advancing personalized medicine approaches for the treatment of neurological disorders.

## Figures and Tables

**Table 1:** Current hPSC-derived astrocyte protocols. hPSC-derived astrocyte differentiation protocols can vary greatly in time, requiring 14 to 250 days to reach functional astrocytes. Protocols can take advantage of 2D and 3D culture methods as well as apply exogenous differentiating compounds or use both exogenous compounds and DOX-inducible transcription factors to activate a gliogenic fate and astrogenesis. As hPSCs are highly sensitive cells, the factors used to expand these cells before or after gliogenic fate choice may play a role in the resulting astrocytes. The scalability of differentiation protocols remains constrained with the “Scalability” column indicating which protocols have confirmed certain aspects of scalability (such as expandability or cryopreservation), and it also points out a protocol for astrocyte differentiation that has been automated.

Reference	Time to Reach Functional Astrocyte	Culture Method	Differentiating Compounds	Scalability
Krencik et al. (2011) [75]	180 days	2D culture—exogenous induction	Expanded with EGF, FGF2. Differentiated with CNTF	1 hPSC: 2.8 × 10^12^ astrocytes, cryopreservable
Tcw et al. (2017) [67]	30 days	2D culture—exogenous induction	Expanded with FGF2. Differentiated with AM ScienCell (FBS)	Expandable, cryopreservable
Sloan et al. (2017) [76]	250–590 days	3D culture—human cerebral cortical spheroids	hSC Neural Induction: FGF2, compound-C, SB-431542, EGF, BDNF, NTS	
Santos et al. (2017) [68]	~56 days	2D culture—exogenous induction	Expanded and differentiated with AM ScienCell (FBS), Noggin, PDGF-AA, FGF2, EGF, FBS, LIF	Expandable, cryopreservable
Li et al. (2018) [73]	52 days	2D culture—DOX inducible transcription factors	Induced transcription factors: NFIA, SOX9. Exogenous Factors: heparin, FGF2, EGF, BMP4 and CNTF	
Canals et al. (2018) [72]	14–21 days	2D culture—DOX inducible transcription factors	Induced transcription factors: SOX9, NF1B. Exogenous factors: FBS, FGF, HB-EGF, CNTF, BMP4	
Tchieu et al. (2019) [71]	60 days	2D culture—DOX inducible transcription factor	Induced transcription factor: NFIA. Exogenous factors: FGF2, EGF, HB-EGF	
Jovanovic et al. (2023) [70]	50 days	2D culture—exogenous induction	Expanded and differentiated with LDN-193189, Jagged-1, DIL-1, Onc-M, PDGF-AA, LIF, CNTF, 1% lipid suppl, hNRG1, forskolin, T3, PMA, Ascorbic Acid	CompacT SelecT robotic cell culture platform: 500 million astrocytes, cryopreservable

Based on final maturation point or time of functional assays. This is not a complete list of all hPSC-derived astrocyte protocols. This list is specific to notable studies mentioned within this review.

**Table 2:** Characterization of hPSC-derived astrocytes in current protocols. hPSC-derived astrocytes undergo varying functional characterizations throughout the literature. This table is meant to visualize which notable studies have verified well-known functional aspects of hPSC-derived astrocytes. Included is whether derived-astrocytes are most closely associated with fetal-like (immature) or adult-like (mature) human astrocytes, which epi/genetic assays and analyses were completed, whose functional characteristics were verified and with what assays or techniques, and a summary of the co-culturing effects of each protocol’s astrocytes.

Reference	Immature or Mature-like	Multi-Omics Datasets	Functional Assays					Co-Culture
			Glutamate Uptake	Phagocytotic Activity	Calcium Transients	Inflammatory Response	Glycogen Storage	
Krencik et al. (2011) [75]	Immature		Patch clamp electrophysiology		Calcium indicator Fluo-4			Neuronal co-culture with derived astrocytes increased astrocytic maturity and neuronal synapses
Tcw et al. (2017) [67]	Immature	RNA-seq		pHrodo red conjugated-myelin and zymosan bioparticles	Calcium indicator Fluo-4AM	Multi-Analyte ELISArray after Abeta42 treatment		Derived-astrocyte co-culture increased the phagocytic activity of BV2 microglia
Sloan et al. (2017) [76]	Mature	scRNA-seq	Radioactive glutamate (L-[2,3,4-3 H] glutamate)	pHrodo red-labeled mouse synaptosomes				Neuronal co-culture with immature and mature astrocytes displayed similar synaptogenic abilities. Mature astrocytes increased Ca+ activity significantly more than immature ones
Santos et al. (2017) [68]	Immature	RNA-seq	Radioactive [3H] glutamate		Calcium indicator Fluo-4	Flow cytometry-based approach and RNA-seq		Neuronal co-culture with inflammatory cytokine stimulated derived-astrocytes resulted in significant decrease in neuronal survival and dendritic length
Li et al. (2018) [73]	Immature	RNA-seq, DNA methylation	Glutamate assay kit		Calcium indicator Fluo-4			iAstro co-culture resulted in greater iN neurite growth, length, and branching
Canals et al. (2018) [72]	Mature		Colorimetric glutamate assay kit		Calcium indicator Fluo-4	RT-qPCR	Basic Fuchsin staining	Co-culture with iAs resulted in greater iN synapse formation
Tchieu et al. (2019) [71]	Immature	RNA-seq, DNA methylation, ATAC-seq			Calcium indicator Fura-2	C3 ELISA Kit, RT-qPCR		Induced-astrocyte co-culture resulted in neuronal protection from excitotoxicity, structural and functional synapses, and decreased resting membrane potential
Jovanovic et al. (2023) [70]	Immature	scRNA-seq, DNA methylation, ATAC-seq	Colorimetric glutamate assay kit		FLIPR Penta high-content calcium imager	C3 ELISA Kit	Periodic acid–Schiff staining	Derived-astrocyte co-culture with neurons resulted in greater neurite length, greater electrical activity, and greater SYP vesicle protein expression as well as greater astrocytic maturation markers

This is not a complete list of all hPSC-derived astrocyte protocols. This list is specific to notable studies mentioned within this review.

## Abbreviations

AD	Alzheimer’s Disease
ALDH1L1	Cytosolic 10-formyltetrahydrofolate dehydrogenase
ALS	Amyotrophic Lateral Sclerosis
AQP-4	Aquaporin 4
AQP-4	Aquaporin-4
APOE4	Apolipoprotein E4
ASD	Autism Spectrum Disorder
ATP	Adenosine triphosphate
BBB	Blood–Brain Barrier
cAMP	Cyclic adenosine monophosphate
Cas 9	CRISPR-associated protein 9
CD44	Cluster of differentiation 44
CNTF	Ciliary Neurotrophic Factor
CRISPR	Clustered regularly interspaced short palindromic repeats
CT-1	Cardiotrophin-1
E	Day of embryonic development of the mouse embryo
EGF	Epidermal Growth Factor
FABP7	Fatty acid binding protein 7/brain lipid binding protein
FDA	United States Food and Drug Administration
FGF-(2)	Fibroblast Growth Factor (2)
FBS	Fetal Bovine Serum
GFAP	Glial Fibrillary Acidic Protein
GLT-1	Glutamate transporter 1
gp130	Glycoprotein Gp 130
IL-1alpha	Interleukin–1 alpha
IL-6	Interleukin-6
iPSC	Induced pluripotent stem cell
JAK	Janus kinases
LIF	Leukemia inhibitory factor
LIFR-β	Leukemia inhibitory factor receptor beta
LRRK2	Leucine rich repeat kinase 2
MS	Multiple Sclerosis
NFIA	Nuclear Factor I-a
NFIB	Nuclear Factor I-b
NG2	Neural/glial antigen 2
NPC	Neural progenitor cell
NCATS	National Center for Advancing Translational Sciences
OPC	Oligodendrocyte Precursor Cell
PD	Parkinson’s Disease
PSEN1/2	Presenilin-1/2
RGC	Radial glia cell
SOX9	SRY-Box Transcription Factor 9
S100B	S100 calcium-binding protein B
SMAD	Small mothers against decapentaplegic intracellular signaling proteins
STAT	Signal Transducer and Activator of Transcription
TDP-43	Transactive response DNA binding protein of 43 kDa
TNF	Tumor Necrosis Factor
